# Modulation of leg trajectory by transcranial magnetic stimulation during walking

**DOI:** 10.1038/s41598-025-05741-3

**Published:** 2025-07-01

**Authors:** Héloïse Bourgeois, Rose Guay-Hottin, El-Mehdi Meftah, Marina Martinez, Marco Bonizzato, Dorothy Barthélemy

**Affiliations:** 1 Institute of Biomedical Engineering, Polytechnique Montréal, Montréal, QC Canada; 2 Electrical Engineering, Polytechnique Montréal, Montréal, QC Canada; 3 Mila, Québec AI Institute, Montréal, QC Canada; 4 Centre de Recherche Interdisciplinaire en Réadaptation (CRIR), Montréal, QC Canada; 5 Département de Neurosciences, Centre Interdisciplinaire de Recherche sur le Cerveau et l’apprentissage (CIRCA) and Centre d’Innovation Biomédicale, Université De Montréal, Montréal, QC Canada; 6 CIUSSS du Nord-de-L’île-de-Montréal, Montréal, Qc Canada; 7 School of Rehabilitation, Université de Montréal, Montréal, Qc H3C 3J7 Canada

**Keywords:** Transcranial Magnetic Stimulation, Locomotion, Foot clearance, Swing phase, Motor cortex, Neurophysiology

## Abstract

The primary motor cortex is involved in initiation and adaptive control of locomotion. However, the role of the motor cortex in controlling gait trajectories remains unclear. In animals, cortical neuromodulation allows for precise control of step height. We hypothesized that a similar control framework applies to humans, whereby cortical stimulation would primarily increase foot elevation. Transcranial magnetic stimulation (TMS) was applied over the motor cortex to assess the involvement of the corticospinal tract over the limb trajectory during human walking. Ten healthy adults (aged 20–32 years) participated in treadmill walking at 1.5 km/h. TMS was applied over the left motor cortex at an intensity of 120% of the threshold to elicit a dorsiflexion of the right ankle during the swing phase of gait. Electromyographic (EMG) measurements and three-dimensional (3D) lower limb kinematics were collected. When delivered during the early swing phase, TMS led to a significant increase in the maximum height of the right toe by a mean of 34.9% ± 9.6% (21.4 mm ± 7.9 mm, p = 0.032) and knee height by 52.8% ± 14.1% (28.8 mm ± 7.7 mm, p = 0.0021) across participants. These findings indicate that TMS can influence limb trajectory during walking, highlighting its potential as a tool for studying cortical control of locomotion.

## Introduction

Rhythmic locomotion is predominantly a spinal process, driven by automated movements even in the absence of conscious control^1^. The initiation, modulation and adaptation of walking are controlled by a complex interplay of multisensory feedback, postural reflexes and supraspinal signals^2–4^. Spinal activity during walking can be actively modulated by cortical circuits, which may be responsible for precise muscle control during human locomotion. The importance of the corticospinal tract (CST) in locomotion and gait recovery has been extensively studied, particularly using techniques like Transcranial Magnetic Stimulation (TMS), a non-invasive technique that involves applying magnetic fields to specific regions of the brain to assess the excitability of cortical and corticospinal pathways^5–7^.

TMS also holds promising potential as a supplementary treatment to current therapeutic protocols. Repetitive TMS (rTMS) has been shown to be effective in improving motor function^8^, reducing neuropathic pain^9^ and alleviating spasticity^10^ in patients with spinal cord injuries.

In terms of locomotor recovery, applying rTMS at high frequencies over the motor cortex of patients with incomplete spinal cord injuries resulted in increased excitability in the CST and led to locomotor improvements^11,12^. Namely, patients who received rTMS showed significant improvements in the Lower Extremity Motor Score, Modified Ashworth Scale, 10-Meter Walk Test, cadence, step length, and Timed Up and Go, compared to those who received sham stimuli. However, these studies did not include kinematic analyses. Thus, the impact of corticospinal excitability on gait trajectories remains unclear. Recent research indicates that applying TMS during robot-assisted walking leads to a reorganization of spinal reflex pathways^13^. However, the artificial nature of this locomotor modality limits kinematic analysis and may restrict the generalizability of findings to natural walking situations.

rTMS is primarily employed to modulate cortical excitability before exercise or task training, with the goal of “priming” motor output and muscle activation. In contrast, the approach adopted in the present study seeks to more directly influence motor cortex output during movement execution, by delivering stimulation in phase with specific gait events, thereby modulating corticospinal signals in a temporally precise manner. The rationale for this targeted, phase-specific strategy is grounded in previous preclinical work. In rats and cats, phasic intracortical neurostimulation—defined as stimulation delivered at specific gait phases—has been used to modulate gait trajectories and coordinate hindlimb movements during locomotion, thus fine-tuning walking patterns^14,15^. Specifically, when stimulation was applied during the swing phase, it reduced foot drop and enhanced flexion capacity. Long-term stimulation-based training promoted long-term recovery of skilled locomotor function in rats with spinal cord injury (SCI)^16^.

A similar approach may hold therapeutic potential for individuals with SCI. However, to our knowledge, no prior study has investigated whether single-pulse TMS, timed to the swing phase of gait, can modulate leg trajectory. Establishing proof of concept in individuals without SCI represents an essential first step toward evaluating the feasibility and potential efficacy of this approach.

Therefore, building on this previous research, our study aimed to understand how cortical networks influence the limb trajectory during swing in healthy humans and to determine whether effects similar to those seen in animal models could be obtained. Although this study uses single pulse TMS and not rTMS, our main hypothesis posits that phase-coherent stimulation over the leg area of the primary motor cortex will increase elevation of the contralateral leg during the swing phase of walking. Our secondary hypothesis states that kinematic effects would vary as a function of the stimulus timing within the swing phase.

## Methods

### Experimental design

The experimental protocol was performed in two parts: first, the hotspot and motor threshold for TMS was determined while the participants were sitting down. These values were then confirmed during quiet standing. In the second part, TMS was applied at different delays during the swing phase to assess its effect on limb trajectory.

### Participants

Ten healthy adults (6 men, 4 women), aged between 20 and 32 years, underwent screening to identify any prior occurrences of seizures, neurosurgical procedures, and the presence of metal or electronic implants, ensuring eligibility for TMS application.

### Ethical approval

The experimental procedures of this study were reviewed and approved by the *Research Ethics Committee en réadaptation et en déficience physique* of the CIUSSS du Centre-Sud-de-l'Île-de-Montréal. The study protocol adhered to institutional guidelines and complied with the principles of the Declaration of Helsinki.

### Informed consent

Written informed consent was obtained from all participants prior to their inclusion in the study. Participants were provided with detailed information about the study objectives, procedures, and potential risks, and they had the opportunity to ask questions before consenting.

### Instrumentation and evaluation

#### Electromyography (EMG)

The EMG signal of the tibialis anterior (TA) and soleus (SOL) muscles was recorded using surface Ag–AgCl electrodes (BlueSensor SP, Ambu A/S, Denmark), 9 mm in diameter and spaced 3 cm apart. After prepping the skin with abrasive tape to ensure it was clean and slightly rough, electrodes were placed on the right TA and SOL muscles, selected for their role in generating locomotor activity at the ankle^17^. The electrodes were placed parallel to the muscle fibers in accordance with SENIAM recommendations^18^. A reference electrode was positioned on the right tibial tuberosity. EMG signals were amplified (× 1000), bandpass filtered (10–1000 Hz), then digitized and sampled at 2 kHz to a computer using a micro1401 interface (Signal software, Cambridge Electronic Design Ltd, UK).

#### Kinematics

A 10-camera Vicon motion analysis system (Ten Vicon Bonita optical cameras, Oxford Metrics, Oxford, UK, and two additional video cameras) with a sampling rate of 100 Hz was used to capture the participants’ movement during treadmill walking. Prior to data collection, 16 reflective markers (14 mm in diameter) were placed on the hip and lower body segments of the participants based on bony landmarks, according to the Plug-in-Gait LowerBody Ai marker set.

#### Temporal parameters of gait

The pressure applied by the feet on the ground was recorded using pressure sensors (Robotshop, CA) positioned under the toes and heels. The sensors under the heels were positioned to detect the initial foot contact to the ground and determine the initiation of the stance phase. Those under the toes were placed to detect the decrease of weight of the toes at the end of stance, and identify the beginning of the swing phase.

### Part 1- Finding hotspot and intensity of TMS during sitting.

**Fig. 1:**
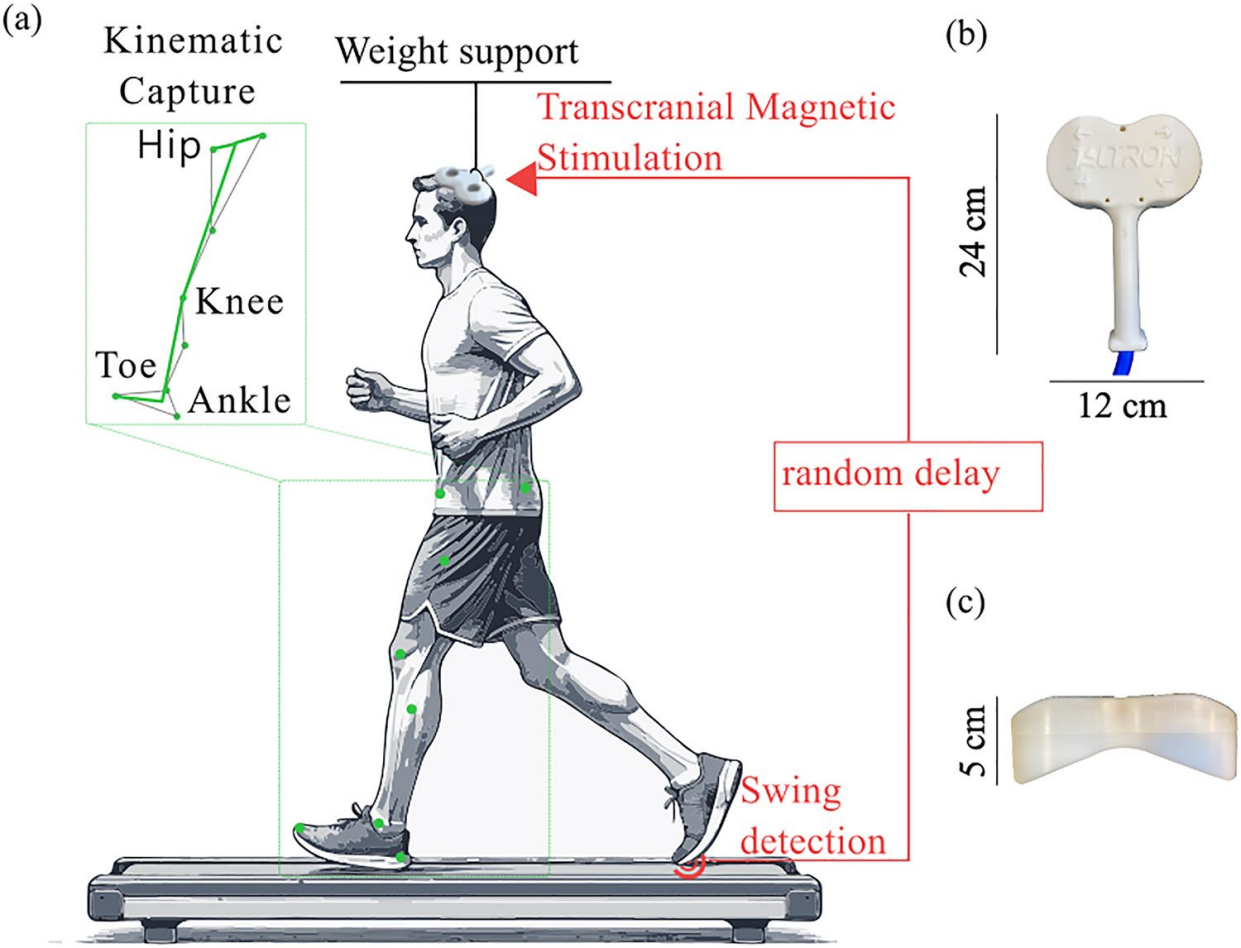
Schematic representation of the experimental setup: (**a**) As the participant walks on a treadmill, TMS is administered via a coil integrated into a helmet, which is weight-supported. Stimulation is triggered by swing detection through the pressure sensor after a randomized delay. Position markers allow for kinematic tracking. (**b**) Top view and (**c**) front view of the custom-made batwing coil used during the experiment.

TMS was applied over the motor cortex during gait using the BiStim2 stimulator (MagStim, UK) and a custom-made batwing coil (Jaltron, US, Fig. 1B). The coil measured 5 cm in height, 12 cm in width, and 24 cm in length. First, in a sitting position, we placed the coil slightly left of the Cz position, using a 45-degree coil orientation angle with respect to the sagittal direction^19^. The intensity was gradually increased until a motor evoked potential (MEP) was elicited in the right TA. Surrounding positions were then mapped at the same intensity to determine the optimal position, referred to as “the hotspot," where TMS induced the largest MEP for the same stimulation intensity^20^. This hotspot position was then recorded as a target in a neuronavigation system BrainSight (Rogue Research Inc., CA) to control for stability of the subsequent stimulation. The neuronavigation system uses optical sensors attached to the participant’s head and the stimulation coil. In real-time, these sensors track the participant’s head movements, allowing for the detection of any deviation from the stimulation target. After setting the coil, the threshold for the MEP and for the dorsiflexion of the ankle was established: Participants performed an isometric contraction of the dorsiflexors corresponding to 10% of their perceived maximal effort while stimuli were applied. The Active motor threshold for the MEP was defined as the minimum stimulus intensity required to elicit a recognizable MEP (over 100 µV) at least 50% of the time, that is in at least 3 out of 5 stimuli^21^. Once this threshold was established, we further increased the intensity to determine the dorsiflexion motor threshold, when a clear dorsiflexion is detected in 3 out of 5 stimuli. We then used an intensity equivalent to 120% of the dorsiflexion motor threshold. The hotspot and intensity of stimulation were validated in the standing position to make sure it induced a measurable dorsiflexion of the right leg before moving to the second part of the protocol, when TMS was applied during gait.

### Part 2- TMS during gait

The participants walked on a treadmill at a speed of 1.5 km/h. Such a slow pace was chosen to generalize to slower walking speeds exhibited by individuals with spinal cord injury^22^ and to enable greater stability for TMS application. Initially, each participant walked on the treadmill for 1–2 min to become accustomed to the speed. Then, the pressure pattern exerted on the sensor under the toe of the right leg was analyzed on-line to identify the level of activity corresponding to the onset of toe-off, when the load on the toe started to decrease. This point corresponded to the onset of the swing phase and constituted the main trigger to determine the timing of application of the stimulation (Fig. 1A).

After the sensors detected the swing initiation, stimuli with a pulse width of 1 ms were administered with delays of 0 ms, 100 ms, 200 ms, 300 ms and 400 ms after toe-off. Twelve stimuli were applied per delay. The order of the stimulation delays was pseudo-randomized, as well as the number of non-stimulated gait cycles between stimulation events, with a minimum of two non-stimulated cycles between each stimulated cycle. The non-stimulated *spontaneous* walking cycles served as a control.

A critical aspect of this setup involved ensuring the stability of the stimulation site amid the rhythmic bodily motions inherent in locomotion while preserving the participant’s comfort and ease of movement. For this purpose, a cyclist’s helmet, modified to provide wide access to the scalp, was utilized. The stimulation coil was mounted on the helmet using adjustable metal fixtures for optimal positioning on the scalp. Due to the heavy weight of the coil (1000 g) and helmet (665 g), they were suspended over the participant’s head using a body-weight support system (Anti Gravity Systems LLC, USA). This relieved the weight of the coil and helmet off the participant’s head throughout the entire session (Fig. 1). Additionally, the neuronavigation system (BrainSight) was used. If a deviation of 6 mm from the target or more occurred, the experiment was halted, and the helmet position was adjusted to restore the optimal positioning.

### Data analysis

We quantified each MEP as the peak-to-peak measurement of the EMG response volley observed 25 ms after stimulation within a 50 ms interval for each stimulus and averaged the MEP amplitude for each stimulation delay. To ensure data comparability across participants, MEP amplitudes were normalized relative to each individual’s largest MEP and expressed as a percentage of that maximum MEP. To evaluate the effects of ongoing EMG levels on MEP amplitude, we calculated pre-stimulus EMG activity for each stimulation interval. A pre-stimulus time interval of 50 ms was selected to measure baseline EMG levels, calculated as the average of the rectified signal over the interval. Similarly, to maintain data consistency across participants, EMG activities were normalized relative to each participant’s largest EMG and the results are presented as a percentage of that maximum EMG.

The 3D camera data were processed using Nexus software, followed by custom software, and then MATLAB (R2022b). Toe-off and heel-strike events were identified from pressure sensor traces to segment the data into gait cycles. We analyzed the vertical, frontal, and lateral excursions in the respective vertical, sagittal, and lateral planes of the toe, knee, and hip during each gait cycle. We also examined the angular excursion, which represents the difference between the maximum and minimum angle of the ankle, knee, and hip in all three planes.

**Fig. 2:**
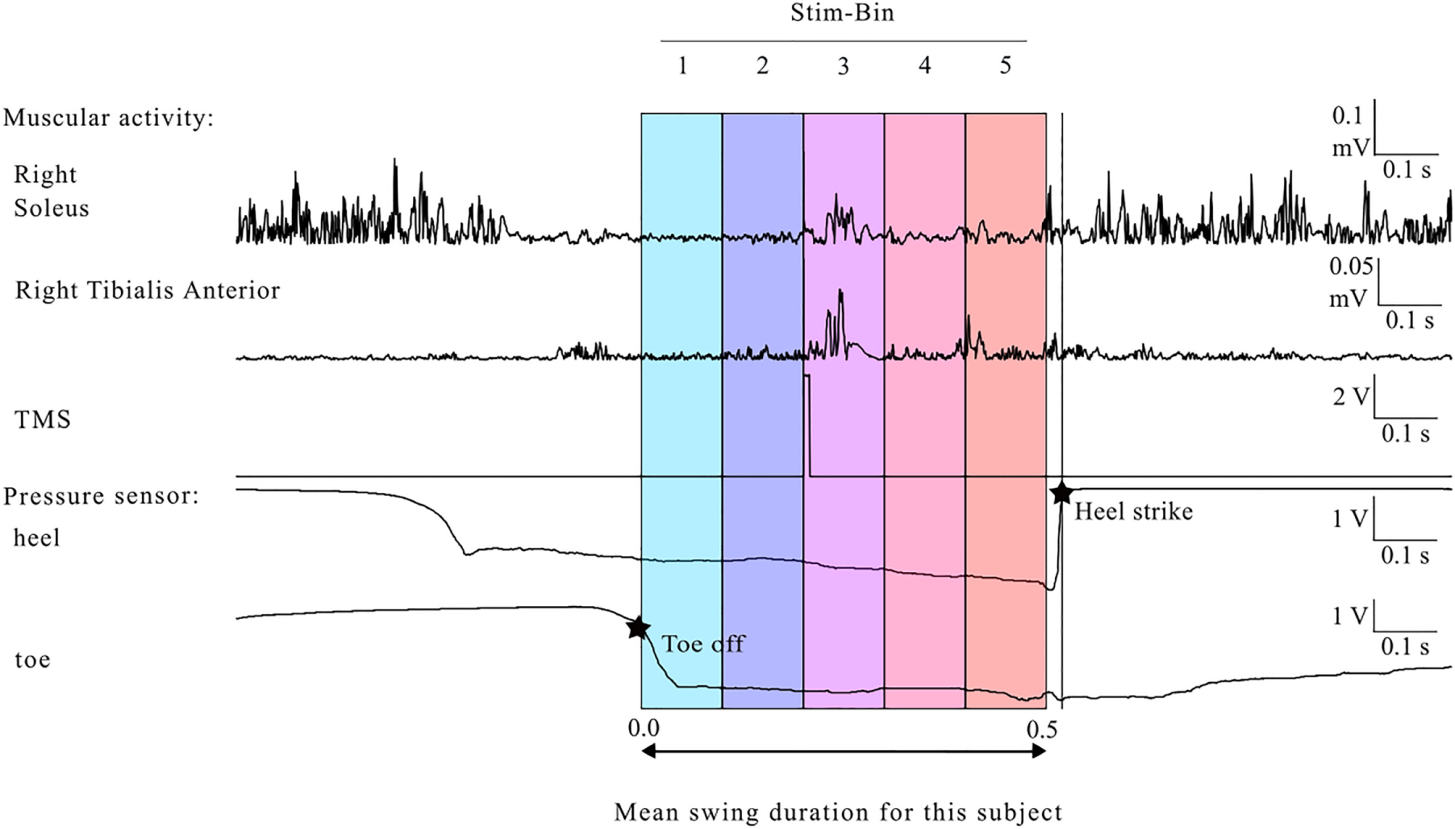
Stimulation labeling based on timing within the swing: Stimulation occurred during the third fifth of the participant’s average swing duration. Consequently, this stimulation is categorized under Stim-Bin3. Tracing of the muscle activity of the SOL and TA, TMS stimulation, and the pressure under the heel and toe. The two stars represent toe-off and heel strike.

Because swing durations varied among participants, fixed-delay stimulations did not always occur in the same swing sub-period. Therefore, to ensure consistency, cycles with stimulation were sorted into five bins according to the time when the stimulation occurred within the swing phase. The average duration of the spontaneous swing phase was divided into five equal time bins. Cycles with stimulation occurring in the first 10% of the cycle were sorted into Stim-Bin1, and so forth (Fig. 2).

### Statistical analysis

To determine the differences in kinematic variables between the non-stimulated cycles and the different Stim-Bins of the stimulated cycles, a one-way repeated measures analysis of variance (RM ANOVA) was conducted. The analysis included ten participants under six different experimental conditions: spontaneous, Stim-Bin1, Stim-Bin2, Stim-Bin3, Stim-Bin4 and Stim-Bin5. Subsequently, a Dunnett post hoc test was performed to compare spontaneous cycles with each Stim-Bin condition.

For the analysis of EMG and MEP data, a one-way repeated-measures analysis of variance was performed to assess differences across the five experimental conditions: Stim-Bin1, Stim-Bin2, Stim-Bin3, Stim-Bin4, and Stim-Bin5. Subsequently, a Tukey post hoc test was performed.

Statistical tests were conducted in GraphPad Prism (v10.2.3). For all statistical tests, significance was set at p < 0.05. Results are presented as mean ± standard error of the mean (S.E.M).

Power analysis: The sample size of n = 10 participants was determined via a power analysis targeting a statistical power of 0.8 (β = 0.2) and a significance level of α = 0.05. For each of the 10 hypothetical participants and for each condition, 10 samples were drawn from a Gaussian distribution (μ =  + 30%, σ = 75%), simulating individual steps per condition. These samples were averaged per participant and condition. A one-way repeated-measures ANOVA followed by a Tukey post hoc test was then applied to the aggregated data. This entire procedure was repeated 10,000 times in a Monte Carlo simulation framework to estimate power. The simulation confirmed that a sample size of 10 participants yields a power of 0.8 at α = 0.05.

## Results

Across all participants, the average number of gait cycles recorded was 225.5 ± 12.9, including 48.6 ± 3 stimulated cycles and 176.9 ± 12 non-stimulated cycles. For Stim-Bin 1 to 4, results average data from 11.3 ± 0.9 cycles per participant. In contrast, Stim-Bin 5 was under-represented, with an average of only 3.6 ± 0.9 cycles per participant. For 20% of the participants, no simulations were classified as Stim-Bin 5, because most of their swing movements were no longer than 400 ms in duration.

### TMS delivered during the early swing modulated the foot trajectory and leg kinematics

**Fig. 3:**
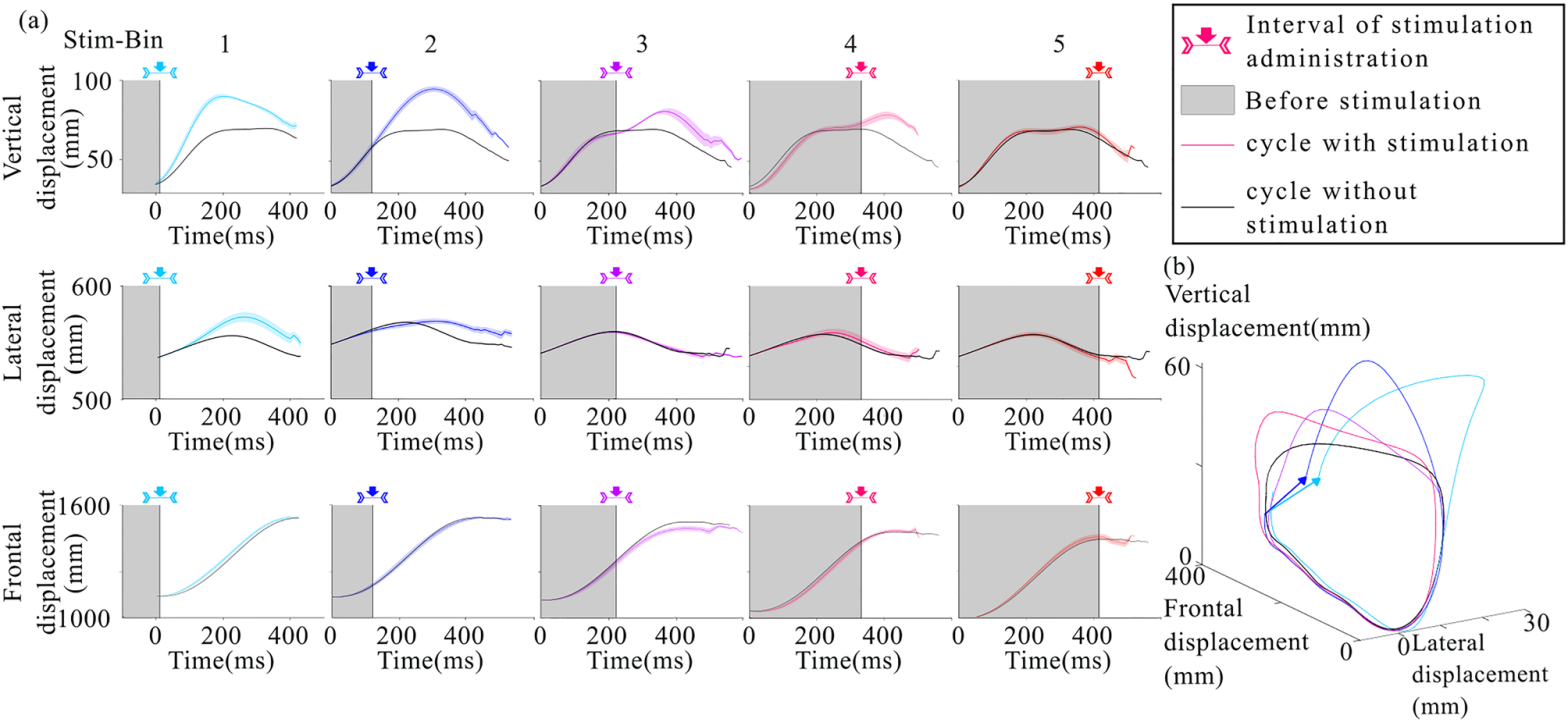
Toe position of a representative participant: (**A**) Average toe position in the vertical, lateral, and sagittal planes for stimulations in each Stim-Bin compared to the spontaneous cycles, (**B**) Average toe position in three dimensions for stimulations in each Stim-Bin and the spontaneous cycles. Arrow indicates displacement of the final foot positioning in Stim-Bin1 and 2.

Figure 3 illustrates the vertical, lateral and frontal displacement of the toe in one representative participant following stimulation in Stim-Bin1 through 5. An increased vertical toe elevation was observed when TMS was applied in Stim-Bins 1 to 4, with no significant effect observed in Stim-Bin5, corresponding to the latter 20% of the swing phase. A larger lateral displacement was also observed in early swing (Stim-Bin1 and 2), and no significant change was observed in the latter part of swing (Stim-Bins 3, 4 and 5). Stimulation did not affect frontal displacement.

**Fig. 4:**
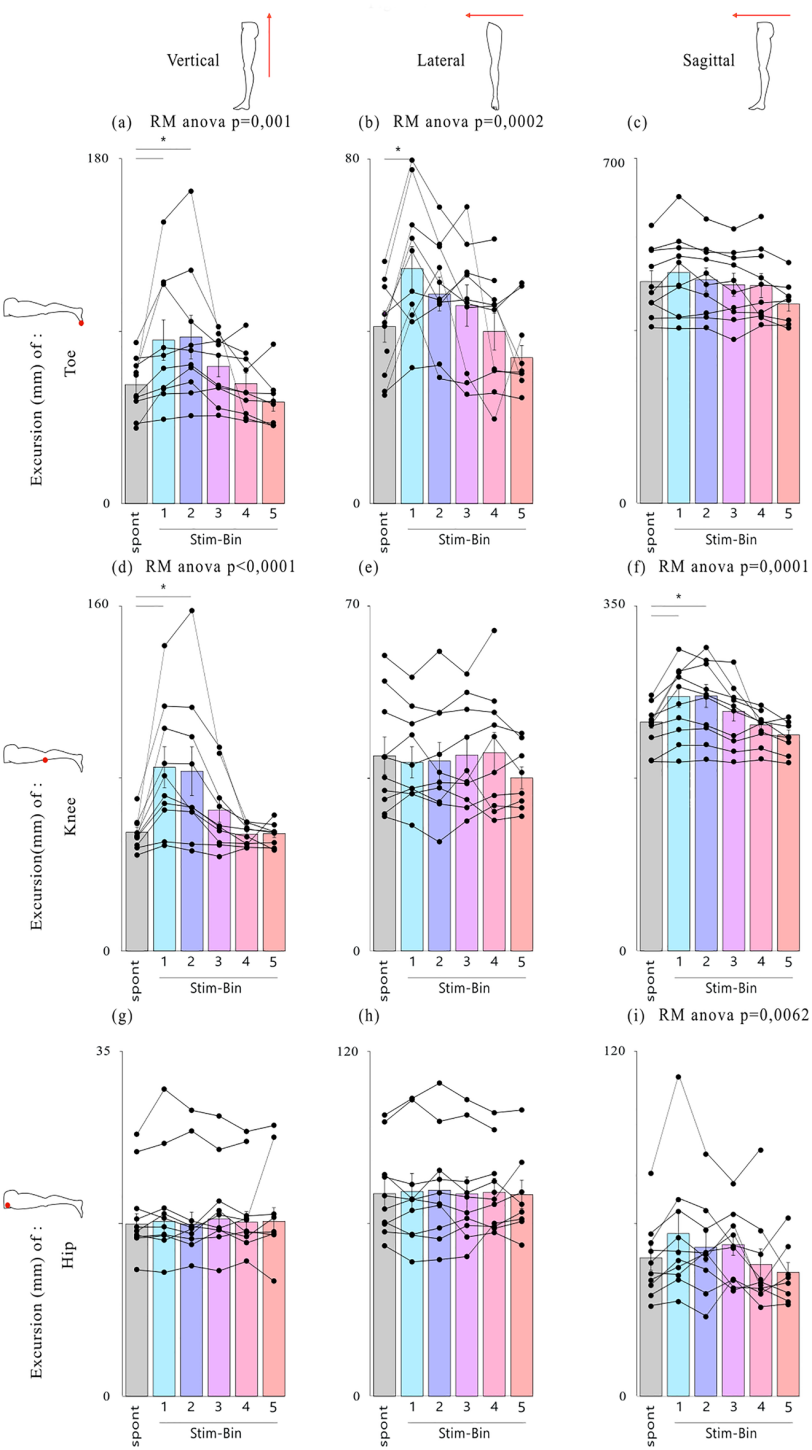
Analysis of leg joint positions for each type of stimulation. Average excursion of the position of the toe(**A**–**C**), knee (**D**–**F**) and hip(**G**–**I**) in the vertical, lateral and sagittal planes, respectively: *p < 0.05, Dunnett’s test, mean ± standard error of the mean (S.E.M).

Similar results were observed across all participants. A significant increase in vertical toe elevation of 34.9% ± 9.6% (21.4 mm ± 7.9 mm, p = 0.032) was observed when stimulation was applied in Stim-Bin1. A similar elevation increase of 39% ± 15.1% (23.9 mm ± 9.2 mm, p = 0.0069) was observed with stimulation in Stim-Bin2, compared to the spontaneous cycles (Fig. 4A). Stimulation in Stim-Bins 3, 4 and 5 did not induce significant toe elevation. Lateral toe excursion also showed a significant increase of 35.1% ± 8.1% (14.4 mm ± 3.3 mm p = 0.0015) when stimulation was delivered in Stim-Bin1 compared to spontaneous cycles (Fig. 4B). No significant differences were observed in the sagittal plane (Fig. 4C). The resulting toe trajectories in all three planes are displayed in Fig. 4A–C.

**Fig. 5:**
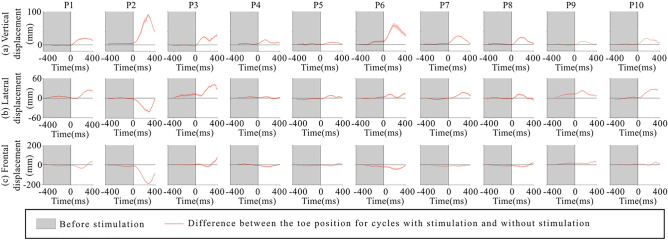
For each participant, the difference between the toe position for cycles with stimulation and without stimulation. (**A**) in the vertical plane, (**B**) in the lateral plane, (**C**) in the sagittal plane. To calculate the displacement, the position of each spontaneous cycle is subtracted from every cycle with stimulation. Thus, the non-shaded portion of the graph represents the average impact of the stimulation immediately after it is delivered.

The mean vertical toe excursion in cycles with stimulation consistently exceeded that in cycles without stimulation, although effectiveness varied among participants. Normalized data for all participants are displayed in Fig. 5. This increase in amplitude ranged from 8.58 mm to 73.8 mm (Fig. 5A). Lateral and frontal toe displacements showed smaller amplitudes than the vertical displacement but displayed variability across participants, notably participant 2 showed opposite lateral displacement (Fig. 5B). ﻿Although most TMS effects were consistent across participants, individual patterns showed reduced frontal toe excursion in cycles with late stimulation (Stim-Bin3, 4, or 5), particularly noticeable in participants 1, 2, and 6, where stimulation caused premature termination of the swing phase before completing the cycle (Fig. 5C).

### Effect of TMS on knee and hip position

Although TMS was optimized to induce activation of the ankle dorsiflexor muscles (namely TA), a significant increase in the vertical knee elevation of 52.8% ± 14.1% (28.8 mm ± 7.7 mm, p = 0.0021) was observed when stimulation was applied in Stim-Bin1. This increase was 49.6% ± 17.2% (27.1 mm ± 9.3 mm, p = 0.0035) when stimulation was applied in Stim-Bin2, compared to spontaneous cycles (Fig. 4D). Furthermore, there was a significant frontal displacement of the knee joint of 10.9% ± 2.4% (24.9 mm ± 6.1 mm, p = 0.0180) when stimulation was delivered in Stim-Bin1 (Fig. 4F). No significant change was observed at the knee joint in the lateral plane (Fig. 4E) or at the hip in any plane (Fig. 4 G, H and I).

### TMS increases ankle and hip angle flexion

**Fig. 6:**
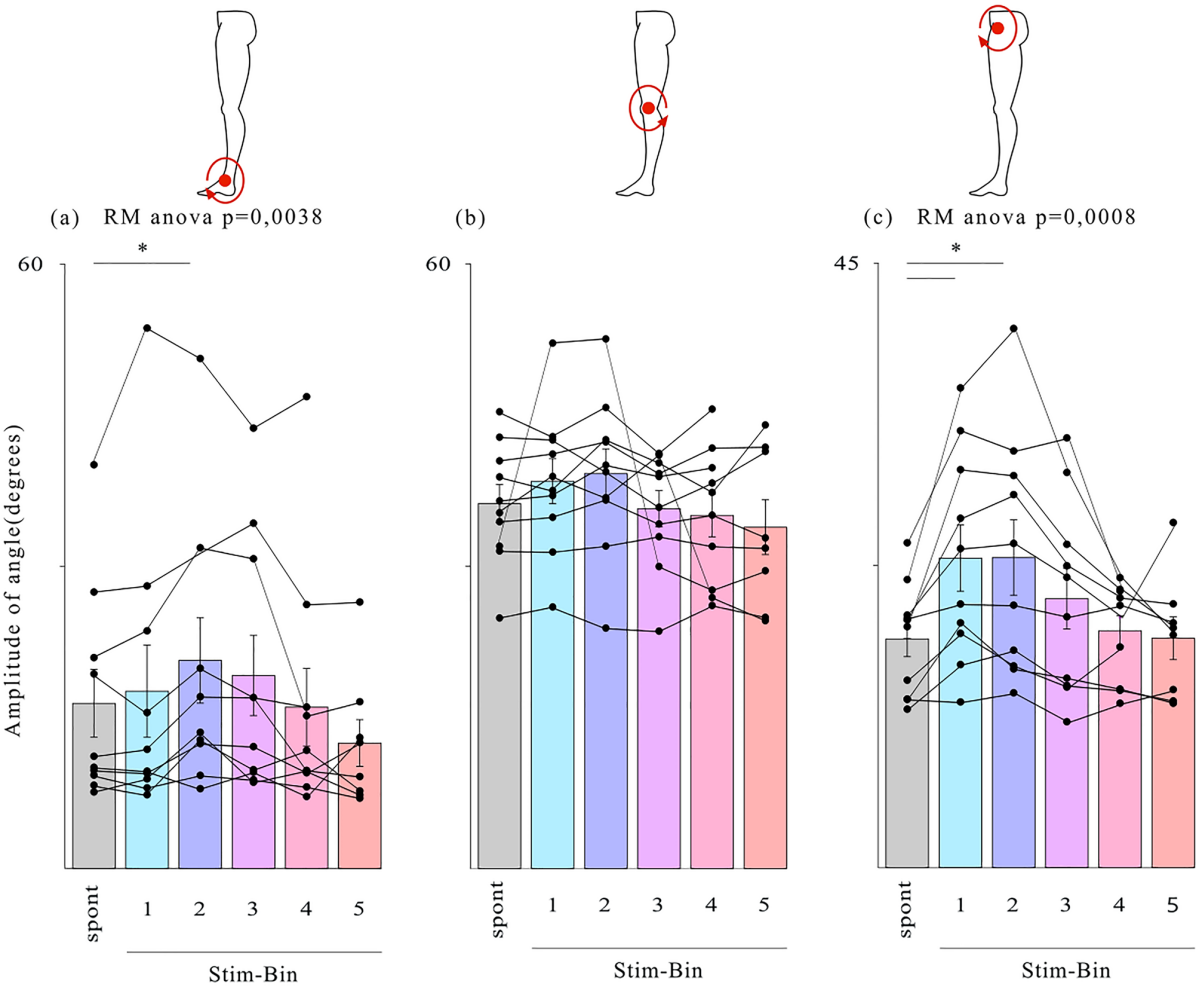
Angular analysis of leg joints for each type of stimulation in the sagittal plane during the swing phase. Average amplitude of the ankle (**A**), knee (**B**) and hip (**C**), *p < 0.05, Dunnett’s test, mean ± standard error of the mean (S.E.M).

Significant increases in angular amplitude during the swing phase (joint flexion, Fig. 6) were observed in the sagittal plane for the ankle and hip joints following TMS. Compared to spontaneous cycles, stimulation delivered in early swing resulted in an increase of 26% ± 8.3% in ankle angular amplitude (4.3 degrees ± 1.4 degrees, p = 0.0362, Stim-Bin2, Fig. 6A) and an increase of 35.3% ± 6.2% in hip angular amplitude (6 degrees ± 1.4 degrees, p = 0.0122, Stim-Bin1, Fig. 6C). There were no significant increases in angular excursion at the knee. Furthermore, no significant differences were observed in the angular amplitude of the ankle, knee, and hip in other planes (Supplementary Figs. 1 and 2).

### Quantification of MEP modulation

**Fig. 7:**
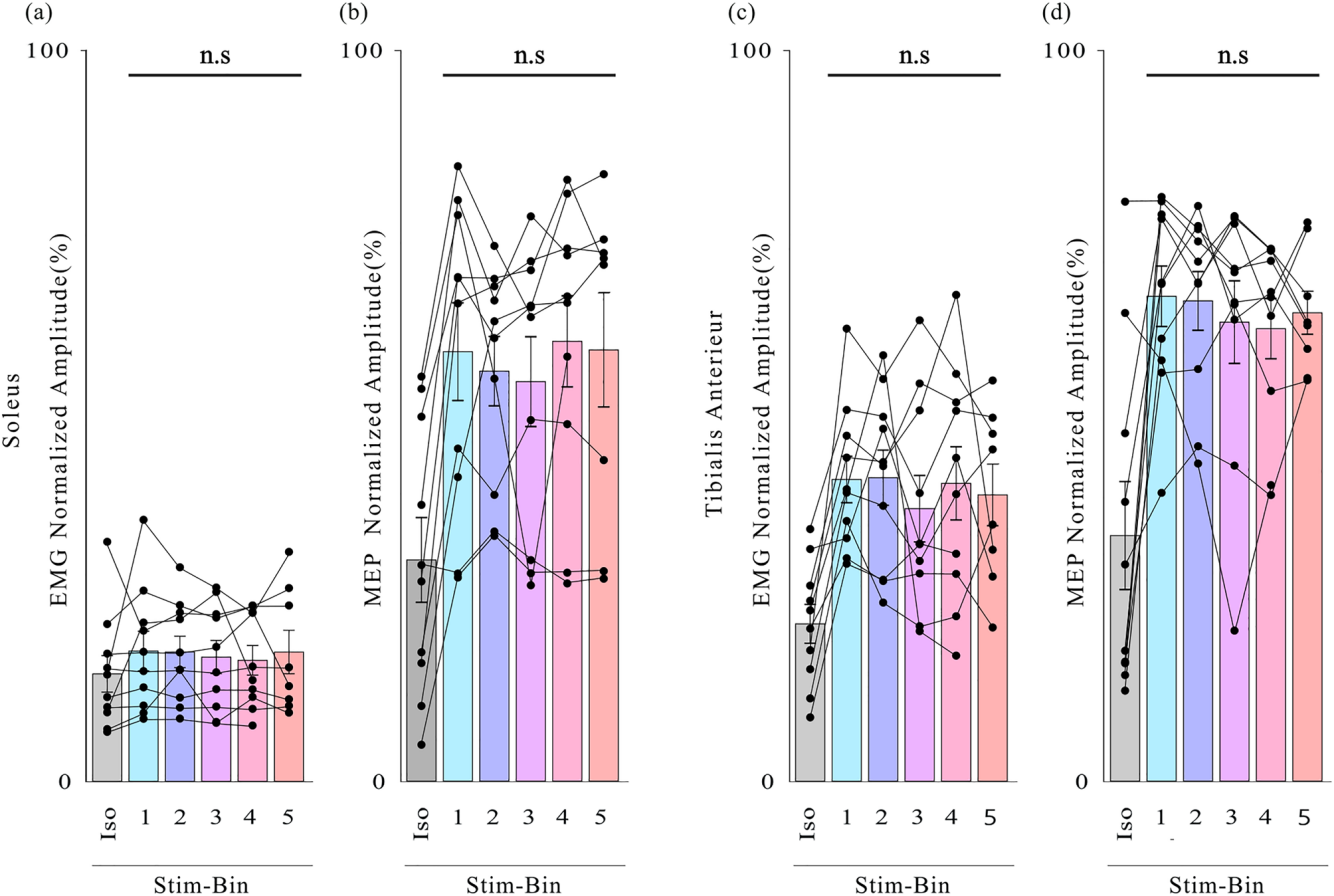
Analysis of muscle activity for each type of stimulation: (**A**) Background EMG amplitudes before stimulation of the SOL, (**B**) MEP amplitude of the SOL, (**C**) Background EMG amplitudes before stimulation of the TA, (**D**) MEP amplitude of the TA. Iso corresponds to isotonic contraction, *p < 0.05, Tukey’s test, mean ± standard error of the mean (S.E.M).

To assess changes in corticospinal excitability in different sub-phases of leg swing, MEP amplitude was recorded for each event of stimulation provided in different Stim-Bins (Fig. 7). Muscle state before stimulation was also evaluated. The SOL did not display changes in activity before stimulation (Fig. 7A), while the TA was most active in early phases of swing (Fig. 7C). Both for SOL (Fig. 7B) and TA (Fig. 7D), no differences were observed between the different Stim-Bins, thus the MEP amplitudes remain constant regardless of the timing of stimulation during the swing phase.

## Discussion

The results of this study highlight the role of the corticospinal tract in controlling foot trajectory during walking, specifically promoting swing elevation. Results show that TMS increased knee and toe elevation when applied at the beginning of the swing phase, albeit with variability among individuals. TMS also induced movements of the knee and toes in the sagittal and lateral directions when applied at the beginning of the swing phase. MEP amplitudes of the TA and SOL were similar in every Stim-Bin during gait, regardless of the timing of stimulation during the swing phase or of the mean level of ongoing EMG in TA during gait. As significant changes in trajectory modulation are observed only in Stim-Bin 1 and 2, modulation of corticospinal excitability to TA and SOL muscles is not correlated to the changes in the trajectory, and more proximal muscles could be preferably recruited by the stimulation during gait.

### TMS leads to increased foot clearance when applied at the onset of swing

The increase in vertical elevation of the toe during swing may be attributed to increased flexion of the ankle, knee and hip, as measured by angular excursion. Despite tuning stimulation amplitude to achieve suprathreshold effects primarily on ankle dorsiflexion (120% intensity), TMS-induced muscle activation involves the entire leg. The human leg motor cortex is located along the edge and depths of the interhemispheric fissure^23^, where TMS can recruit more superficial cortical networks^24^. Networks of neurons more selectively controlling distal leg movements, particularly the ankle, are located deeper within the fissure, potentially requiring different stimulation techniques for recruitment. The preferential recruitment of flexor muscles aligns with previous findings in the cat model, which have demonstrated a predominance of cortical control over flexor muscles. One study^25^ reported that intracortical stimulation during locomotion evoked larger responses and often recruited additional muscles compared to stimulation at rest. Specifically, stimulation during the swing phase increased flexor muscle activity without changing the cycle duration, whereas stimulation during the stance phase decreased extensor muscle activity duration and initiated a new, premature swing phase, thereby resetting the step cycle. Thus, activation of the cortical and corticospinal tracts preferentially recruits flexor muscles, which might be located more proximally. As trajectory is most changed when TMS is applied in early swing, one potential target for TMS might be proximal leg flexor muscles. Indeed, in the cat model, one study recorded discharges from the pyramidal tract neurons within the hindlimb representation of the primary motor cortex^25^. Many pyramidal tract neurons became active early in the swing phase, at the same time as muscles like the semitendinosus that help bend the knee. Others fired a bit later, in sync with ankle flexors such as the tibialis anterior, and some became active toward the end of swing, alongside muscles that lift the toes. One limitation of our study is that knee flexor or hip flexor muscles were not recorded, which prevents a clear conclusion on this point. Future investigations should look more closely at proximal flexor muscles of the lower limb. We also did not analyze TMS effects on the ipsilateral leg, nor did we attempt to calibrate TMS delivery to muscles other than the TA. Thus, modulation of alternative recruitment patterns and ipsilateral leg responses remains unexplored. A further limitation of using TMS during walking is that coil fixation to the head always involves some positional tolerance. Although we monitored coil displacement with a tracking system and halted the experiment if displacement exceeded 6 mm, even smaller shifts may have impacted the accuracy of hotspot positioning. Such shifts could have influenced the reported effect size, which nonetheless remained significant across our ten participants. Since much of our focus is on foot endpoint trajectory during swing, our data were collected only during the swing phase. Thus, conclusions cannot be extended to potential postural changes that could be induced by delivering stimulation during the stance phase. Previous studies suggest that corticospinal involvement in flexor activation occurs throughout the gait cycloxy_comment_ende^26^, and cortical contributions during stance have also been demonstrated, notably through reductions in soleus EMG following cortical suppression^5^.

### TMS induces changes in sagittal and lateral trajectories of knee and toe

The induced lateral and frontal changes in the trajectories of knee and toe suggest that TMS not only recruits neuronal networks controlling flexor muscles but also neighbouring networks that control slightly different movements. Although these movements were not targeted, they might not be inconsistent with the ongoing walking task. In healthy individuals^27^, an abduction of the ankle and an external rotation of the hip during the swing phase contribute to a lateral external displacement of the toe^28^, which was observed in the current study. Similarly, in healthy individuals, it is observed during walking that knee flexion during the swing phase results in lateral knee displacement, which was observed in the present study, and is further amplified by TMS stimulation. Invasive microstimulation studies in intact cats have shown that intracortical microstimulation induces various gait patterns, including lateral abductions and diverse foot lifts biased toward the front or back^14^. Outputs from neighboring regions can linearly summate^29^, suggesting that individual movement outcomes depend on the recruited subregions within the effective cortical activation area, coil positioning, and individual cortical configuration and excitability.

### Constant MEP amplitudes in TA regardless of timing of the stimulation

The consistency of MEP amplitude indicates that the timing of TMS within the gait cycle does not differentially affect corticospinal excitability. This might suggest a stable level of cortical excitability throughout the gait cycle or that MEPs are not sensitive to the specific timing of TMS in this context. This may seem to contrast with previous studies showing strong MEP modulation induced by TMS during walking, likely reflecting changes in cortical excitability^26^. Nonetheless, MEP amplitude in TA stayed constant in each Stim-Bin despite significant trajectory modulation only in the 1^st^ and 2^nd^ Stim-Bin. This suggests that TA may not be the primary agonist of this trajectory modulation and, as stated above, TMS might preferentially recruit proximal flexor muscles at the hip and knee that can enable this early change in limb trajectory. These results are in line with studies in rats with spinal cord injury: Specifically, neurostimulation applied to the motor cortex effectively lifts the foot during the swing phase of locomotion, involving the entire leg and being most effective when applied early in the swing phase^16^. Furthermore, in the rat model, the intensity of intracortical neuromodulation correlated with the step height, indicating precise control over leg kinematics. Our observations in humans using TMS as cortical neuromodulation confirm these results and point to similar underlying mechanisms, such as cortical neuronal adaptations, adjustments in neural circuits governing locomotion, and coordinated muscular responses to fine-tune lower limb movements. While MEPs elicited by single-pulse TMS are widely interpreted as indicators of corticospinal excitability, it is important to recognize that they do not exclusively reflect cortical activity. Modulations in MEP amplitude may also arise from changes in the excitability of subcortical structures, as well as at the spinal level. Moreover, MEP amplitudes were normalized to the largest MEP obtained across all conditions. While the gold standard for normalization is the maximal M-wave (Mmax), obtaining Mmax at each tested delay would significantly increase data collection time and reduce the feasibility of the protocol. Normalizing to the largest MEP is unlikely to alter the correlations or differences observed between the bins, but it may increase variability at the group level, as reflected by larger standard errors.

## Conclusion

This study demonstrates that TMS can significantly influence limb trajectory during gait by increasing flexion during the swing phase. TMS most likely also recruits neuronal networks controlling flexor muscles located at proximal joints, as no corresponding changes in corticospinal excitability in ankle flexors were observed during gait. These findings shed light on mechanisms enabling the cortex and corticospinal tract to influence the limb trajectory during gait. Furthermore, the current study introduces an innovative application of TMS: applying it during walking to adjust lower limb trajectory and improve foot elevation.

## Supplementary Information

Supplementary Information 1.

Supplementary Information 2.

Supplementary Information 3.

## Data Availability

All data generated or analysed during this study are included in this published article and its supplementary information files.

## Abbreviations

TMS: Transcranial Magnetic Stimulation
EMG: Electromyographic
rTMS: Repetitive Transcranial Magnetic Stimulation
MEP: Motor Evoked Potential
SCI: Spinal cord injury
TA: Tibialis Anterior
SOL: Soleus
3D: Three-dimensional
Mmax: Maximum M-wave
