# Supplementary figures and images for: Modulation of leg trajectory by transcranial magnetic stimulation during walking
Source: Sci Rep. 2025 Jul 1;15:21362. doi: 10.1038/s41598-025-05741-3 (PMC12215949; doi:10.1038/s41598-025-05741-3)

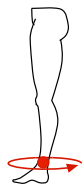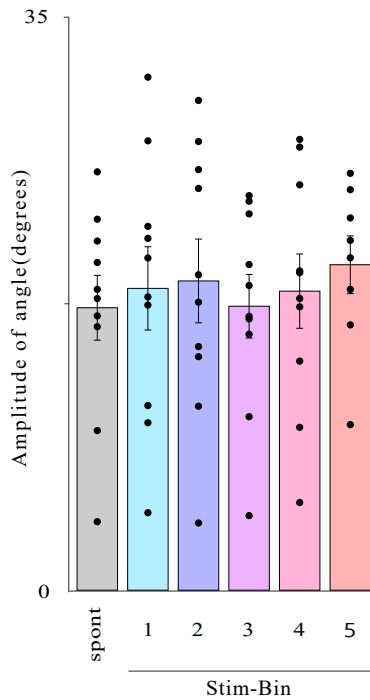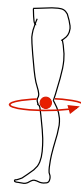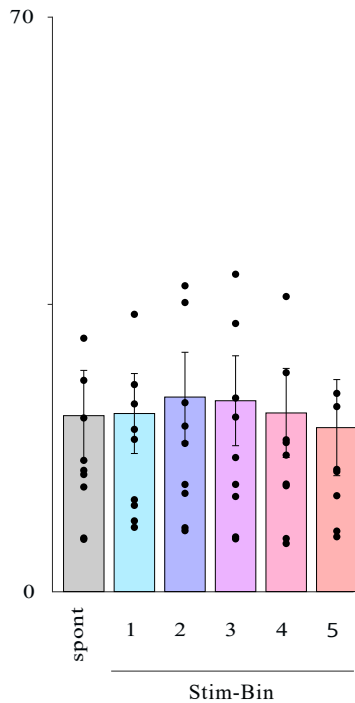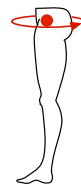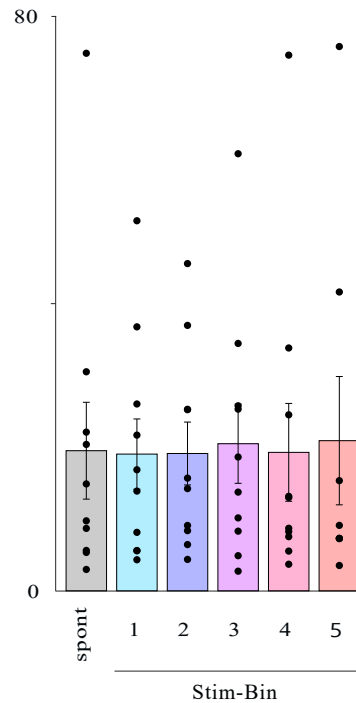

Supplement: Supplementary file 1 — Supplementary Information 1. [file 41598_2025_5741_MOESM1_ESM.pdf]

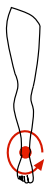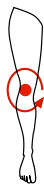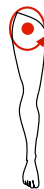

RM anova  $p=0,0225$

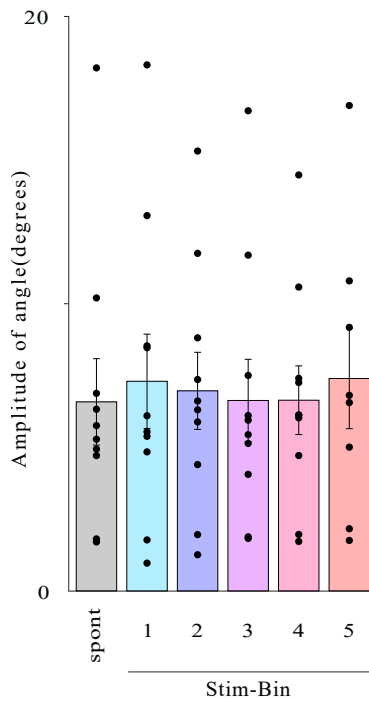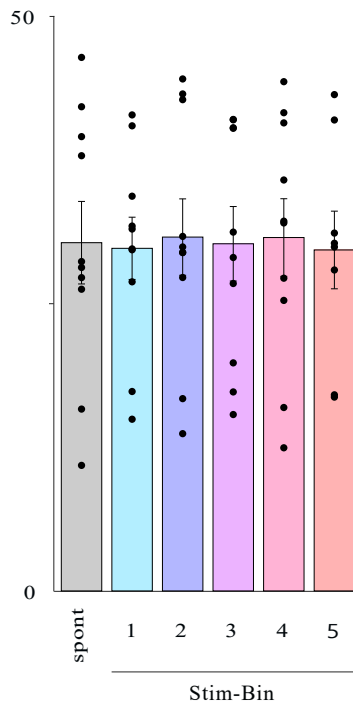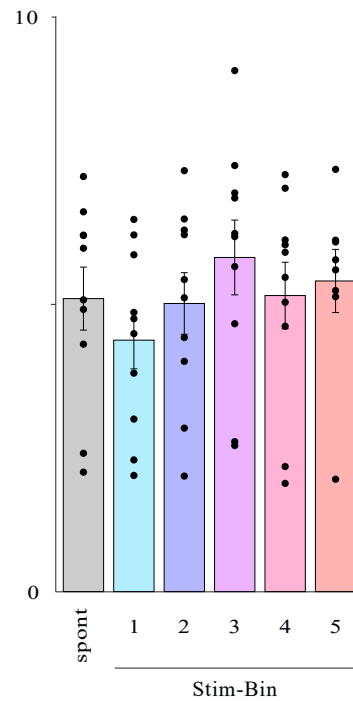

Supplement: Supplementary file 2 — Supplementary Information 2. [file 41598_2025_5741_MOESM2_ESM.pdf]
